# An Unusual Case of Impending Paradoxical Embolism Through a Patent Foramen Ovale in Renal Cell Carcinoma

**DOI:** 10.7759/cureus.34964

**Published:** 2023-02-14

**Authors:** Bhanu P Maturi, Ashwini Sharma, Kenneth J Wool, Zeyad Elmarzooky, Varshitha Kondapaneni

**Affiliations:** 1 Internal Medicine, University of Alabama at Birmingham (UAB), Montgomery, USA; 2 Cardiology, Baptist Medical Center South, Montgomery, USA; 3 Cardiology, Heart of the Rockies Regional Medical Center, Salida, USA; 4 Cardiology, University of Alabama at Birmingham (UAB), Montgomery, USA

**Keywords:** pulmonary embolism, impending paradoxical embolism, thrombectomy, renal cell carcinoma, patent foramen ovale

## Abstract

Impending paradoxical embolism (IPDE) is a clinical emergency with adverse outcomes. Due to its rarity, larger research cannot be obtained to provide definitive therapy alternatives. We report a case of a tumor embolus from a renal cell carcinoma (RCC) that caused a right atrial mass, pulmonary embolus, and impending paradoxical embolus via a patent foramen ovale (PFO) and its management.

## Introduction

Paradoxical embolism represents an uncommon condition that occurs when a thrombus originating from the venous system results in systemic embolization through an intracardiac or pulmonary shunt [1]. The most frequent cause of an intracardiac shunt is a patent foramen ovale (PFO). Depending on the site of the embolism, patients present with varied presentations [2]. Impending paradoxical embolism (IPDE) is described as the presence of a thrombus straddling through PFO [3]. It constitutes a clinical emergency due to the risk of massive systemic embolization.

## Case presentation

**Figure 1:**
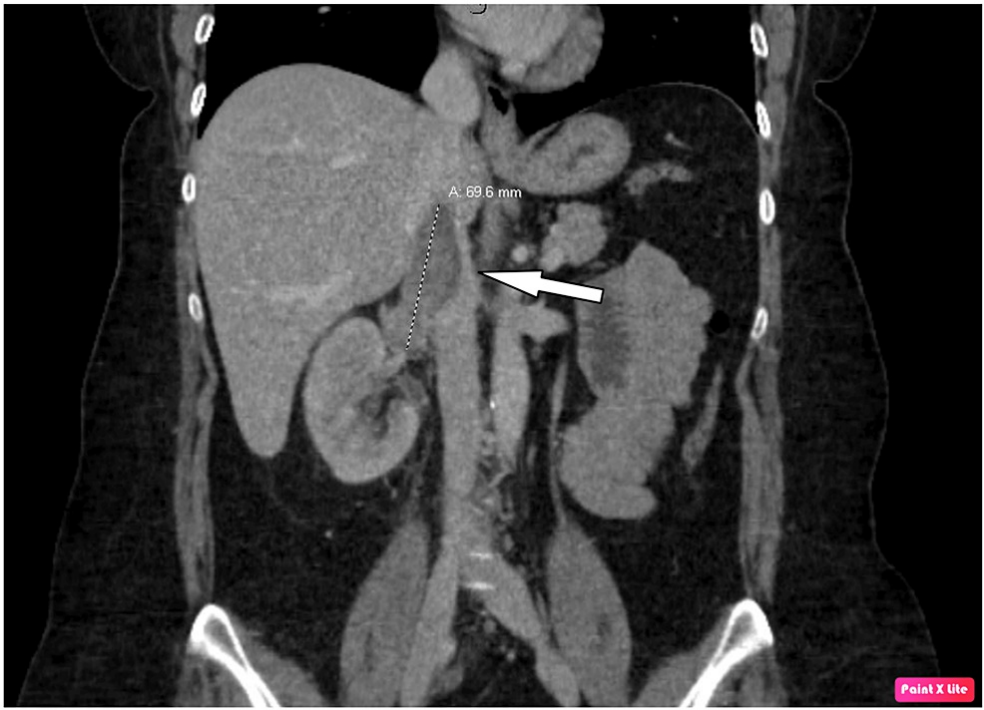
CT of the abdomen and pelvis showing a right renal mass with inferior vena cava extension (white arrow) CT: computed tomography

The patient is a 48-year-old Caucasian female who presented to the emergency room (ER) with acute right flank pain and hematuria. Computed tomography (CT) of the abdomen and pelvis showed a right renal mass with extension to the inferior vena cava (IVC) (Figure 1) and retroperitoneal lymphadenopathy.

**Figure 2:**
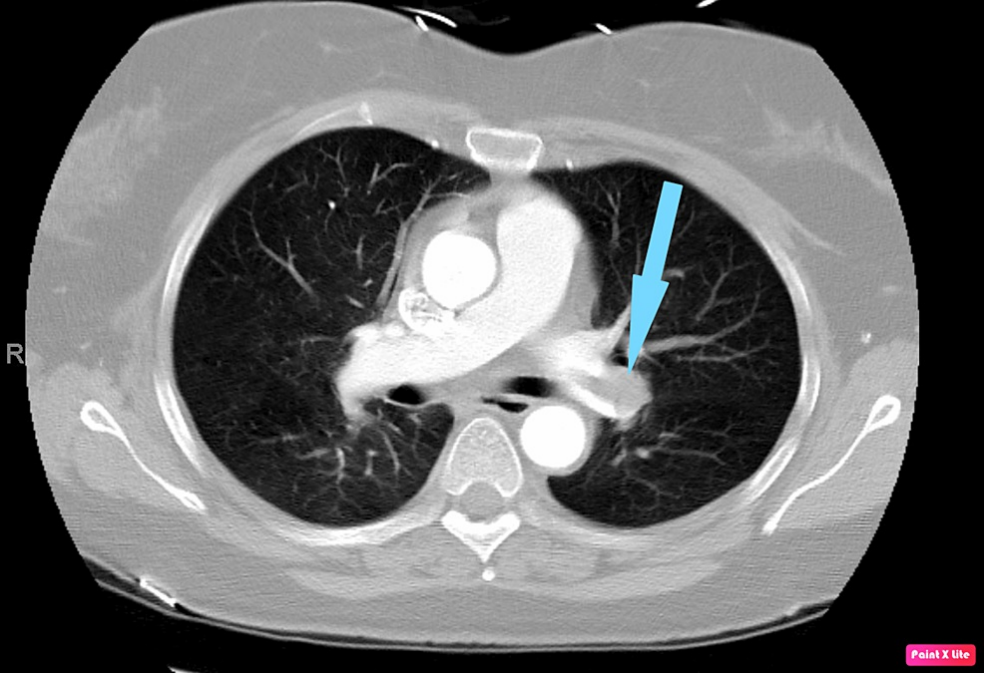
Thoracic CT showing left pulmonary artery embolus (blue arrow) CT: computed tomography

**Figure 3:**
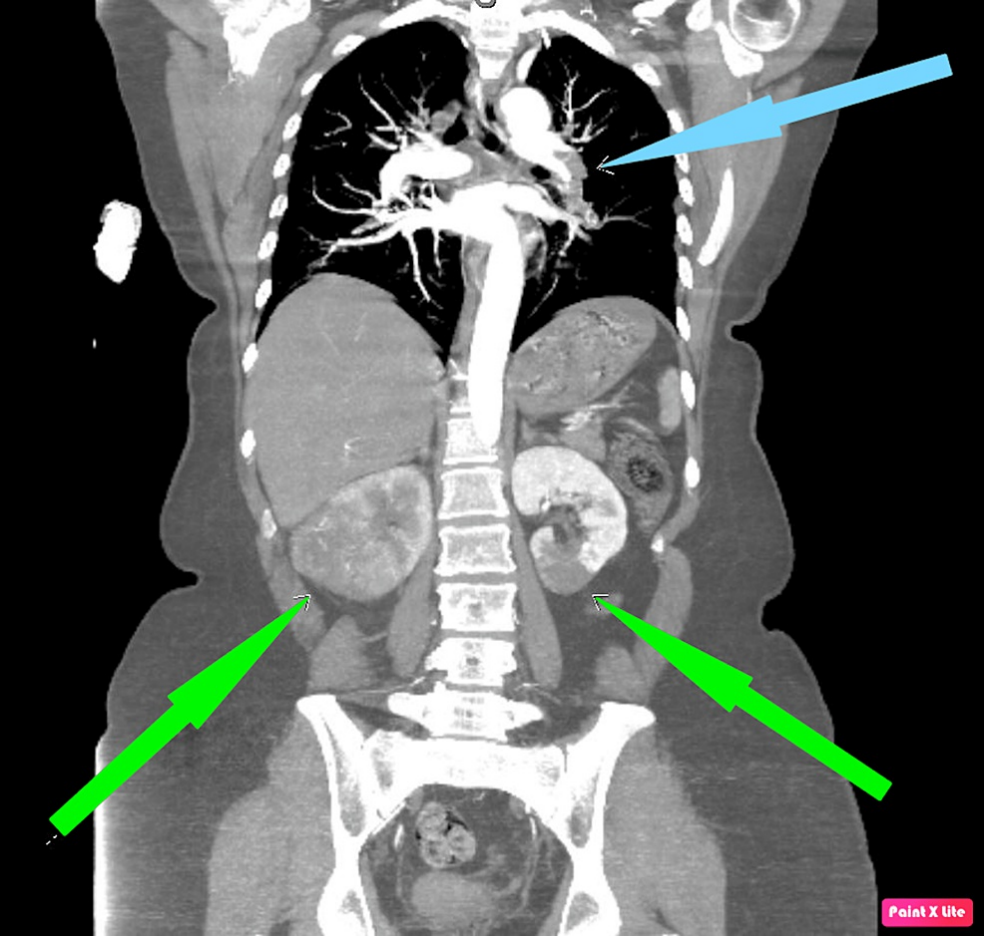
CT of the abdomen and pelvis showing left pulmonary embolus (blue arrow) and bilateral renal infarcts (green arrows) CT: computed tomography

All other diagnostic workups had unremarkable results. The patient has an extensive history of smoking and a family history of renal cell carcinoma (RCC). She was scheduled for an elective radical nephrectomy within three days. However, after two days, she returned to the ER due to shortness of breath and an episode of syncope. Physical examination revealed that she was tachycardic and in moderate respiratory distress. CT angiography of the chest showed a submassive pulmonary embolus (Figure 2), and CT of the abdomen and pelvis with contrast showed right and left renal infarcts (Figure 3).

These findings were not present during her previous ER visit. The cardiology service was consulted to evaluate the possibility of aspiration thrombectomy for pulmonary embolism. Transesophageal echocardiography (TEE) was performed for further evaluation of renal infarcts as there was a concern of systemic embolization. TEE revealed the presence of a dense and large mobile mass across the PFO (Video 1 and Video 2). CT surgery was consulted. The patient was consequently admitted to the intensive care unit. Then, she underwent a nephrectomy, IVC thrombectomy, pulmonary embolectomy, and PFO closure. Histopathologic results revealed that the surgical samples from the right atrium and pulmonary arteries consisted of clear cell RCC and thrombi. The postoperative course was unremarkable, and she recovered without any adverse events. She was discharged with subcutaneous enoxaparin.

**Video 1:** TEE four-chamber view showing an echo-dense mass in transit through the PFO TEE: transesophageal echocardiography, PFO: patent foramen ovale

**Video 2:** 3D TEE bicaval view showing an echo-dense mass in transit through the PFO 3D: three dimensional, TEE: transesophageal echocardiography, PFO: patent foramen ovale

## Discussion

A paradoxical embolism is a condition that occurs when a thrombus originating from the venous system leads to systemic embolization through an intracardiac or pulmonary shunt [1]. Based on the site of embolization, paradoxical embolism may have varied presentations, such as ischemic stroke [2], myocardial infarction, acute abdomen due to bowel ischemia, hematuria from renal infarction, or peripheral arterial occlusion. IPDE is described as the presence of thrombotic material straddling the PFO [3]. The first case of paradoxical embolism was reported in 1877, but the first case of IPDE was reported only in late 1985 [4,5]. IPDEs are often not visualized by transthoracic echocardiogram. TEE is a very crucial tool in diagnosing IPDE. If a paradoxical embolism with a shunt is suspected, an emergency TEE should be conducted to rule out IPDE.

IPDE is a medical catastrophe without emergent treatment. Because of its rarity, large-scale studies could not be done to provide definitive therapy alternatives. Treatment modalities include surgical thrombectomy, systemic thrombolysis, and anticoagulation. Percutaneous interventions are not recommended in this condition, at least because of the risk of systemic embolism and high mortality. Systemic thrombolysis has a similar mortality when compared with surgical thrombectomy until 2005. Since 2005, surgical thrombectomy has been associated with lower mortality and also fewer posttreatment embolization events [6]. This is primarily due to the advancement of surgical techniques or improvements in perioperative management. Systemic thrombolysis is still considered in patients who are unstable and also with severe comorbidities [7]. This situation serves to support the notion that surgery is the optimum course of treatment for patients with IPDE who are hemodynamic, as opposed to thrombolytic therapy, which should only be utilized in patients who are unstable and cannot tolerate surgery [8].

## Conclusions

An impending paradoxical embolism is a condition where definitive treatment strategies are hard to be made due to its rarity. Because it is associated with a worse prognosis, it should be taken into consideration in all paradoxical embolism patients. Although systematic reviews have some limitations, this case supports surgical thrombectomy as superior to systemic anticoagulation or thrombolysis in stable patients.
